# Editorial: The Challenge of New Therapeutic Approaches for Unmet Therapeutic Needs

**DOI:** 10.3389/fphar.2020.01341

**Published:** 2020-09-30

**Authors:** Arianna Carolina Rosa, Mitsunobu Mio, Ioanna Andreadou, Vadim V. Sumbayev

**Affiliations:** ^1^ Department of Scienza e Tecnologia del Farmaco, University of Turin, Turin, Italy; ^2^ Department of Pharmacology, School of Pharmacy, Shujitsu University, Okayama, Japan; ^3^ Laboratory of Pharmacology, Faculty of Pharmacy, National and Kapodistrian University of Athens, Athens, Greece; ^4^ Medway School of Pharmacy, Universities of Kent and Greenwich, Chatham, United Kingdom

**Keywords:** pharmacology innovation, drug-design, innovative therapies, pharmaceutical innovations, efficacy/risk ratio

In light of the recent worldwide emergency caused by the coronavirus disease (COVID-19), it has become even more urgent that we solve unmet medical needs (UMNs). The COVID-19 pandemic has placed national health systems, as well as scientific research communities under enormous pressures in terms of drug repositioning, which is considered to be the quickest possible transition from bench to bedside for UMNs (Singh et al., 2020). Conceived before the pandemic occurred, this Research Topic is topical and relevant to this context but does not fully address the pitfalls of COVID-19 management. As many working in this area are aware, the larger the disease burden, the greater the degree to which important needs are not met. Consistently, clinical testing and the approval of new medicines involves an accelerated authorization procedure. According to the article 4 paragraph 2 of the Commission Regulation EC (No. 507/2006 of 29 March 2006), the conditional authorization of medicinal products for human use (falling within the scope of Regulation, EC No. 726/2004 of the European Parliament and the Council UMN) takes place when there is “a condition for which there exists no satisfactory method of diagnosis, prevention or treatment in the Union or, even if such a method exists, in relation to which the medicinal product concerned will be of major therapeutic advantage to those affected”.

This topic is focused around a review by Scavone et al. entitled “The New Paradigms in Clinical Research: From Early Access Programs to the Novel Therapeutic Approaches for Unmet Medical Needs”, an extended review which describes the regulatory framework and the more importantly, criticism of new drugs covering UMNs. The other 16 papers in this Research Topic include reviews and research papers which cover an apparent and high heterogeneity of subjects. This collection aims to reflect on a number of important UMNs in different medical areas, to address the lack of a real consensus on the definition and interpretation of UMN (Vreman et al., 2019). Consistently, a search on PubMed.gov using the keyword “unmet therapeutic needs” retrieves 5,100 results, with a variety of specific therapeutic areas that reflect the complexity of the topic. The above definition of UMN includes the concept of “satisfactory method” or, in other words, “adequate treatment” and “major therapeutic advantage”, also meaning “significant benefit”. This implies: (i) a degree of disease severity or burden; (ii) a lack of effective drugs; (iii) the existence of non-responders among the treated patients; (iv) poor adherence to the prescribed regimen affecting the extent of concordance or compliance in medicine taking, and therefore conditioning the pharmacotherapy response; (v) the need for safer drugs; and (vi), that the route of drug administration is eventually related to the delivery formulation, to improve both the pharmacokinetic and the compliance in medicine taking. These are the main areas covered by the papers collected in the present Research Topic.

One of the areas where the UMNs are evident is oncology. The burden of cancer and mortality is associated with a need to face up to these different challenges (Smith et al., 2015). This Research Topic includes several exemplary studies. For instance, the paper “Transcriptome-Wide Effects of Sphingosine Kinases Knockdown in Metastatic Prostate and Breast Cancer Cells: Implications for Therapeutic Targeting” by Alshaker et al., which studied oncogenic signaling to explore the potential advantages of certain molecular therapy combinations in targeting prostate and breast cancers, which reveals the importance of the RNA transcriptome microarray analysis.

A research snapshot article by Sakhnevych et al. on “Mitochondrial Defunctionalization Suppresses Tim-3-Galectin-9 Secretory Pathway in Human Colorectal Cancer Cells and Thus Can Possibly Affect Tumor Immune Escape”, further indicates the importance of understanding oncogenic signaling and how it controls immune escape. This enables medical practitioners to select the correct therapeutic targets and develop new approaches for immunotherapy of cancer.

Personalized medicine emphasizes the importance of predicting which patients can experience the best benefits, as well as in anticipating adverse effects. A paper from Giri and Aittokallio on how “DNMT Inhibitors Increase Methylation in the Cancer Genome” approaches this topic, identifying 638 novel hypermethylated molecular targets that influence responses to DNA methyltransferase inhibitors. The synergic effect of different compounds to improve the long-term survival of oncologic patients subjected to conventional chemotherapy is another topic of interest. Accordingly, a prototype for a novel antileukemic therapy is described by Trachtenberg et al., exploring the “Synergistic Cytotoxicity of Methyl 4-Hydroxycinnamate and Carnosic Acid to Acute Myeloid Leukemia Cells via Calcium-Dependent Apoptosis Induction”.

The topic of dose reduction, as a way of limiting multiple the side effects of chemotherapeutic regimens, is critical to solving at least part of the UMNs in oncology. Paclitaxel together with other microtubule inhibitors, such as nocodazole and vinorelbine dose-response curves, were investigated by Potashnikova et al. in the study “Non-linear Dose Response of Lymphocyte Cell Lines to Microtubule Inhibitors”, who demonstrate, at the cellular level, how the dose can influence the clinical response in terms of cell cycle arrest and cell death. The development of new formulations, and how these are applied to oncology is also reported. The paper on “Paclitaxel-Loaded Nanosponges Inhibit Growth and Angiogenesis in Melanoma Cell Models” by Clemente et al. assess the problem of how an already existing agent, like paclitaxel, could increase the positive efficacy/risk ratio through an innovative technological approach, based on nanoformulation.

A wide spectrum of nanoformulations is constantly under investigation, to address the need for improving the pharmacological and therapeutic properties of drugs (Bamrungsap et al., 2012). Apart from the study of paclitaxel-loaded nanosponges cited above, an example of functionalized gold nanoparticles is also described by Yasinska et al. The paper “Targeting of Basophil and Mast Cell Pro-Allergic Reactivity Using Functionalised Gold Nanoparticles”, discuss how gold nanoparticles can be used as a platform for the non-toxic delivery of signaling inhibitors, circumventing the side effects of toxic agents such as calcineurin inhibitors, and thus furnishing potential new antiallergic drugs based on a disease-modifying approach (targeting allergic effector cells).

The review articles by Ingusci et al. on “Gene Therapy Tools for Brain Diseases“ and by Chazot et al. on “Histamine and Delirium: Current Opinion” focus the attention on another therapeutic area known to present a number of UMNs, the area of cognitive disorders and other central nervous system disorders, including neurodegenerative diseases. The specific topic of brain disease is very complex and wide. The UMNs in this context are related to unique biological/pathological mechanism(s) that reflect the many as yet unaddressed facets of diseases (Smith et al., 2019). Therefore, as described by Ingusci S. and colleagues, gene therapy represents an attractive option that could address this gap. This Research Topic reports a paper on the search for new antiepileptic drugs is reported, however, an exhaustive exploration of the UMNs for brain disease would require a dedicated issue in itself. Another paper on how “Embelin Prevents Seizure and Associated Cognitive Impairments in a Pentylenetetrazole-Induced Kindling Zebrafish Model” by Kundap et al. discusses the development of a new drug, embelin (produced by *Embelia ribes*), a potential candidate against chronic epilepsy-related cognitive dysfunction with an improved efficacy/risk ratio (retarding seizure and improving cognitive impairment).

This Research Topic also includes another approach to different therapeutic areas such as pathological bone resorptive diseases, osteoporosis, periodontitis, and rheumatoid arthritis. Outlining the novel imidazole derivative KP-A038, Ihn et al. describe the “Inhibitory Effect of KP-A038 on Osteoclastogenesis and Inflammatory Bone Loss Is Associated With Downregulation of Blimp1”. Durante et al. also write about the “Effects of PARP-1 Deficiency and Histamine H4 Receptor Inhibition in an Inflammatory Model of Lung Fibrosis in Mice”. Their focus on lung fibrosis, is based on the new target identification approach, expanding this concept to the possibility of a combination strategy to improve the benefit/risk ratio adding the selective inhibition of the histamine H4 receptor to non-toxic doses of selective PARP-1 inhibitors.

This idea of the identification of a new potential target to solve pitfalls in the effectiveness of existing therapies reflects the review “Glucocorticoid-Induced Leucine Zipper: A Novel Anti-inflammatory Molecule” by Bereshchenko et al., which provides a clear and complete description, focusing on how a consolidated therapeutic area like inflammation, should involve the design of new therapeutic approaches to improve the glucocorticoids benefit/risk ratio.

According to the WHO, cardiovascular diseases (CVDs) are the leading cause of death worldwide. Therefore, CVDs represent another important area in which UMNs persist. Two papers approach this specific topic: the one by Huang et al. entitled “Rolipram, a PDE4 Inhibitor, Enhances the Inotropic Effect of Rat Heart by Activating SERCA2a” and another by Loi et al. on how “Metformin Protects the Heart Against Hypertrophic and Apoptotic Remodeling After Myocardial Infarction”. The first used the prototype of phosphodiesterase-4 inhibitors to validate this target in developing agents for the treatment of heart failure. The second one examined the opportunity of a drug repositioning approach in the treatment of hypertrophic remodeling after myocardial infarction. As an approach, the latter could have important clinical benefits in reducing the experimental time and is based on a well-known commercially available drug-like metformin in the specific.

Some research on UMNs examines polychemical medical approaches, such as those used in Traditional Chinese Medicine, as a possible way to fill that gap (Cheng, 2015). In the paper “Epigoitrin, an Alkaloid From Isatis indigotica, Reduces H1N1 Infection in Stress-Induced Susceptible Model in vivo and in vitro” by Luo et al., this approach is validated by the consistency of preparation, evidence-based clinical efficacy, safety, and knowledge of the mechanism of action.

In conclusion, this Research Topic (containing 3 review articles, 1 mini-review, 3 brief research reports, 1 research snapshot, and 9 original research papers) explores and exemplifies the main challenges in approaching the UMNs. These include: (i) the identification of disease etiopathology and the underlying determinant molecules; (ii) the development of new drugs against novel targets or agents with higher selectivity for existing targets to improve the positive efficacy/risk ratio of the available therapeutic options; and (iii), new strategies for drug administration that could offer better clinical outcomes.

## Author Contributions

ACR conceived and wrote the text. MM, IA, and VVS co-edited the Research Topic and approved the Editorial final version. All authors contributed to the article and approved the submitted version.

## Funding

This work was supported by Fondo Finanziamento delle Attivita Base di Ricerca - ROSA_FFABR_17_01.

## Conflict of Interest

The authors declare that the research was conducted in the absence of any commercial or financial relationships that could be construed as a potential conflict of interest.
